# The modulation of potassium channels by estrogens facilitates neuroprotection

**DOI:** 10.3389/fcell.2022.998009

**Published:** 2022-10-26

**Authors:** Xian-Tao Li

**Affiliations:** ^1^ School of Medicine, Guizhou University, Guiyang, China; ^2^ Department of Neuroscience, South-Central University for Nationalities, Wuhan, China

**Keywords:** estrogens, neuroprotective effects, K^+^ channels, therapeutic benefit, apoptosis

## Abstract

Estrogens, the sex hormones, have the potential to govern multiple cellular functions, such as proliferation, apoptosis, differentiation, and homeostasis, and to exert numerous beneficial influences for the cardiovascular system, nervous system, and bones in genomic and/or non-genomic ways. Converging evidence indicates that estrogens serve a crucial role in counteracting neurodegeneration and ischemic injury; they are thereby being considered as a potent neuroprotectant for preventing neurological diseases such as Alzheimer’s disease and stroke. The underlying mechanism of neuroprotective effects conferred by estrogens is thought to be complex and multifactorial, and it remains obscure. It is well established that the K^+^ channels broadly expressed in a variety of neural subtypes determine the essential physiological features of neuronal excitability, and dysfunction of these channels is closely associated with diverse brain deficits, such as ataxia and epilepsy. A growing body of evidence supports a neuroprotective role of K^+^ channels in malfunctions of nervous tissues, with the channels even being a therapeutic target in clinical trials. As multitarget steroid hormones, estrogens also regulate the activity of distinct K^+^ channels to generate varying biological actions, and accumulated data delineate that some aspects of estrogen-mediated neuroprotection may arise from the impact on multiple K^+^ channels, including Kv, BK, K_ATP_, and K_2P_ channels. The response of these K^+^ channels after acute or chronic exposure to estrogens may oppose pathological abnormality in nervous cells, which serves to extend our understanding of these phenomena.

## Introduction

Estrogens, a group of sex steroid hormones that are majorly synthesized in the ovary, serve an important role in tuning physiological and pathophysiological functions of multiple tissues, such as the heart and brain. In the clinic, therefore, hormone replacement therapy (HRT) with estrogens exhibits numerous beneficial influences on postmenopausal women, including reducing cardiovascular incidence (Mendelsohn and Karas, 2007), protecting cognitive functions (Brinton, 2001), and preventing the occurrence of osteoporosis (Ikeda et al., 2019). Nevertheless, clinical studies reveal that the administration of estrogens is capable of enhancing the risk of breast, ovarian, and endometrial cancer (Cheskis et al., 2007), with several of the biggest negative effects of this therapy substantially threatening woman’s health. In order to avoid the adverse effects of HRT on the human body, selective estrogen receptor modulators (SERMs), such as tamoxifen and raloxifene, have been developed to impact selective target cells or tissues, exerting the therapeutic benefits without the proliferative action (Cheskis et al., 2007).

It is well established that two identified subtypes of nuclear estrogen receptors (ERs), ERα and ERβ, are responsible for estrogen-induced biological effects on distinct tissues. In the classical model, ERs bind to estrogen response elements (EREs), which reside in the promoters of specific genes, and subsequently regulate the related gene expression (Björnström and Sjöberg, 2005). Apart from the genomic actions, membrane-associated ERs enable rapid non-genomic actions, thereby activating protein-kinase cascades (Lösel and Wehling, 2003) or tuning other targets such as K^+^ channels (Tanabe et al., 1999).

A great deal of evidence indicates that the normal functions of the brain, such as emotion, memory, and cognition, are noticeably influenced by sex steroid estrogens throughout life (Taxier et al., 2020). Interestingly, these ovarian hormones impact not only female but also male sexual behaviors by modulating neuronal functions (Taziaux et al., 2007; Taxier et al., 2020). Clinical data have revealed that treatment with estrogens leads to the improvement of menopause-related depressive symptoms (Toffol et al., 2015); moreover, neuroprotective effects are substantially evident after estrogen-containing hormone therapy by researchers, which exhibits a positive action in the broad spectrum of nervous malfunctions including cognitive aging and brain injury (Engler-Chiurazzi et al., 2017). Estrogens conduct multiple actions on the structure and function of the hippocampus, a key brain region for learning and memory. These hormones are able to generate enhanced long-term potentiation by increasing dendritic spine density and the ratio of NMDA transmission to AMPA transmission in the hippocampal neurons (Smith and McMahon, 2005). ERβ activation can boost the dendritic branching and density of mushroom-type spines in these neurons, resulting in improving hippocampus-dependent cognition (Liu et al., 2008). The critical role of the hippocampus in mediating the memory-enhancing effects of estrogen is well established (Tuscher et al., 2019). Estrogen deficiency leads to enhanced hippocampal autophagy and promotes apoptosis in the hippocampus, consequently inducing memory loss (Pandey et al., 2020). Modulation of the hippocampal function by estrogens contributes to the estrogenic facilitation of learning and memory through diverse aspects, such as intracellular cascade, dendritic spinogenesis, and synaptic function and plasticity, and it protects hippocampal cells against excitotoxicity arising from drugs (Taxier et al., 2020). 17β-Estradiol (E2), an isomer of estradiol that is the most potent estrogen, is able to induce upregulation of the expression of neuroprotectant neuroglobin (NGB) to counteract H_2_O_2_-induced apoptosis (De Marinis et al., 2013; Barreto et al., 2021). Exposure to 17β-estradiol also protects the brain against reperfusion injury after acute ischemic insults by reducing lesion formation and brain atrophy, as well as improving cerebral blood flow in rat ischemic stroke (Carpenter et al., 2016). In global ischemia, treatment with 17β-estradiol prevents the loss of CA1 hippocampal neurons in response to ischemic injury (Shughrue and Merchenthaler, 2003), and neuroprotection of estrogen on hippocampal neurons can be achieved by enabling ERα and ERβ (Miller et al., 2005). On the other hand, neuron-derived 17β-estradiol (E2), which is produced locally by the neurons and which is essential for governing synaptic plasticity and cognitive function (Lu et al., 2019), also displays neuroprotective effects through induction of astrocyte activation, which in turn facilitates the release of the neurotrophic factors, BDNF and IGF-1, and the clearance of neurotoxic glutamate after ischemic brain injury (Lu et al., 2020). Collectively, the accrued evidence provides insight that the neuroprotective actions conferred by estrogens are mediated in multiple ways, including antagonizing inflammatory injuries, Aβ toxicity, and oxidative insults, as well as maintaining mitochondrial membrane integrity and calcium homeostasis (Simpkins et al., 2009). Apparently, the interaction of estrogens with multiple targets is needed to support the profound role of this female hormone in protecting the nervous system, and a growing body of evidence has suggested that the K^+^ channel is as a critical mediator involved in the neuroprotective benefits induced by estrogens, providing an alternative mechanistic explanation.

K^+^ channels that selectively conduct K^+^ ions are ubiquitously distributed in non-excitable and excitable tissues, with the latter including cardiomyocytes, neurons, and pancreatic β-cells, and they serve crucial roles in regulating a variety of biological processes, such as proliferation, apoptosis, cellular volume, and secretion (Shieh et al., 2000). In the central and peripheral nervous system, K^+^ channels, especially voltage-gated K^+^ (Kv) channels, are important to set the resting membrane potential, shape the action potential, and tune the firing frequency, contributing to neuronal excitability, synaptic transmission, and neurotransmitter release (Shah and Aizenman, 2014). It is well established that K^+^ channels are closely associated with varying neurological disorders, and dysfunction of these channels can participate in the development of human neurological disease, such as Alzheimer’s disease (AD) and epilepsy (Djillani et al., 2019; Villa et al., 2020). For instance, the mutated KCNQ2 and KCNQ3 genes, which encode Kv7.2 and Kv7.3, respectively, enable benign familial neonatal convulsions (Maljevic and Lerche, 2014), and genetic suppression of small-conductance Ca^2+^-activated K^+^ channels (SK) in deep cerebellar neurons can result in ataxia (Lam et al., 2013). Investigators consider K^+^ channels as a therapeutic drug target for these neurological abnormalities (Humphries and Dart, 2015), and there are even existing clinical studies of treating epilepsy with a drug targeting K^+^ channels (Shieh et al., 2000).

Indeed, experimental results have indicated that K^+^ channels are a key protein for participation in the development of distinct neurological pathologies, indicating a possible role of these channels for neuroprotection. Oxidation of Kv2.1 channels, a major component of the delayed rectifier K^+^ current in neurons (Murakoshi and Trimmer, 1999), leads to enhanced channel activity, and more effluxes of K^+^ ions can in turn promote caspase activation and apoptosis (Shah and Aizenman, 2014). In Alzheimer’s disease (AD), the most prevalent age-related neurodegenerative disorder with progressive impairment of cognitive function and memory, the malfunction of Kv channels is referred to as an essential factor that is relevant to the pathogenesis of AD. Neurotoxic Aβ peptides enable the elevated expression of Kv3.4 and Kv4.2 channels, accounting for A-type K^+^ currents (IK_(A)_) and accordingly triggering neuronal apoptosis (Pan et al., 2004; Pannaccione et al., 2007). Hence, researchers may assume that a promising neuroprotective manner against proapoptotic stimuli serves to prevent K^+^ efflux in nerve tissues (Yeh et al., 2020). On the other hand, excitotoxicity mediated by ischemic or epileptic insults results in the boost of K^+^ efflux by activating Kv channels such as Kv2.1 channels, with a hyperpolarizing shift of voltage-gated activation (Aras et al., 2009), to mitigate the excitability in cultured rat cortical neurons. As a clinical medicine, the Kv7 channel agonist retigabine, which can limit the neuronal excitability, is approved by the FDA to treat epilepsy (Amabile and Vasudevan, 2013). Considerable evidence also reveals that K^+^ channels are emerging as one of the multitargets of estrogens known as potent neuroprotectants, which is indicative of a possibility in which modulation of K^+^ channels may contribute to the neuroprotective actions of these steroid hormones in the nervous system. This review summarizes the reported observations whereby the application of estrogens generates an impact on varying K^+^ channels, which may allow an alternative delineation concerning the involvement of K^+^ channels in estrogen-mediated neuroprotective effects.

## Kv channels

Voltage-gated K^+^ (Kv) channels, consisting of six transmembrane domains and cytosolic N- and C-terminal domains, are the largest and most complex family. Kv channels widely expressed in many types of neural tissues serve to participate in the repolarization of action potentials and govern the firing rates, thereby tuning neuronal excitability and synaptic transmission (Norris et al., 2010). Emerging evidence implies that exposure to estrogens could have an impact on diverse subtypes of Kv channels, such as Kv1.5, Kv2.1, Kv2.2, Kv4.2, and Kv4.3, and corresponding native currents, to produce genomic and non-genomic effects.

Kv1.5 channels, underlying the ultra-rapid rectifier K^+^ current (IKur) in cardiac atria, are located in nervous tissues such as the hippocampal and cortical neurons, and they also conduct the delayed rectifying K^+^ current (IK(_DR)_) in the brain (Maletic-Savatic et al., 1995). Lowering the expression of Kv1.5 channels confers a decrease in neuronal injury and death in ischemic stroke (Stenzel-Poore et al., 2003). Treatment with estrogens results in the downregulation of Kv1.5 transcripts (Tsang et al., 2004; Saito et al., 2009), but no direct inhibition on this channel is observed (Wong et al., 2008) that is indicative of a genomic action of the sex hormones. In line with the data, tamoxifen, a selective estrogen receptor modulator (SERM), conducts an enhanced expression of Kv1.5 channels through blocking the estrogen receptor (ER) (El Gebeily and Fiset, 2011).

As slowly inactivating K^+^ channels, Kv2.1 channels conduct the major delayed rectifier K^+^ current in the cortical and hippocampal neurons (Murakoshi and Trimmer, 1999), holding a fundamental role in shaping intrinsic neuronal excitability. Apart from those expressed in nervous tissues, the distribution of Kv2.1 channels is evident in diverse cell subtypes, such as pancreatic β-cells (MacDonald and Wheeler, 2003), cardiomyocytes (Nerbonne and Kass, 2005), and smooth muscle cells (Schmalz et al., 1998), exhibiting varying effects in regulating physiological functions. Extensive studies have established a clear role of Kv2.1 for modulating neuronal excitability (Speca et al., 2014); furthermore, this channel is also thought to be a crucial regulator of neuronal apoptosis in a number of neural cells in response to diverse apoptotic stimuli, including oxidative stress, ischemic injury, and Aβ toxins (Pannaccione et al., 2007; Sesti et al., 2014; Shah and Aizenman, 2014). Hence, Kv2.1 channels are closely associated with neuroprotective effects owing to their ability to regulate the excitability and apoptosis of neurons. Acute exposure to 17β-estradiol significantly blocks Kv2.1-dominated outward Kv currents in human osteoblast-like MG63 cells and Kv2.1 channels reconstituted in COS-7 cells, with an IC_50_ of 0.3 μM (Li et al., 2013). Corresponding with this report, Kv2.1- and Kv2.2-mediated Kv currents are also inhibited by acute application of 17β-estradiol in the medial preoptic nucleus (MPN), with an EC_50_ value of 9.7 μM (Druzin et al., 2011). Although the normal levels of estrogens are less than 10 nM in mammals (Naftolin et al., 1990), the concentrations of estrogens in target tissues could reach the range of micromolar as the result of an accumulation effect (Noe et al., 1992; Clarke et al., 2001). In addition to rapid non-genomic effects, the genomic regulation induced by estrogens is manifested in a reverse way in that expression of Kv2.1 channels is increased because of blockage of estrogen receptors by tamoxifen (El Gebeily and Fiset, 2011). Consistent with the notion of Kv2.1 inhibition in favor of anti-apoptotic effects (Sesti et al., 2014), therefore, the acute and chronic inhibitory actions of Kv2.1 channels conferred by estrogens may contribute to their neuroprotective influence. Moreover, the decrease in expression of Kv2.1 channels induced by tacrine, an acetylcholinesterase (AChE) inhibitor, leads to a boost in the proliferation of N2A cells, an alternative way to protect neuronal cells (Hu et al., 2020). Another member of the Kv2 family, the Kv2.2 channel, is also abundantly expressed in a variety of neuronal subtypes, and resveratrol, a naturally occurring phytoalexin, produces a blocking effect on Kv2.2 channels through the estrogen receptor GPR30-mediated PKC pathway in cerebellar granule cells (Dong et al., 2013).

**FIGURE 1:**
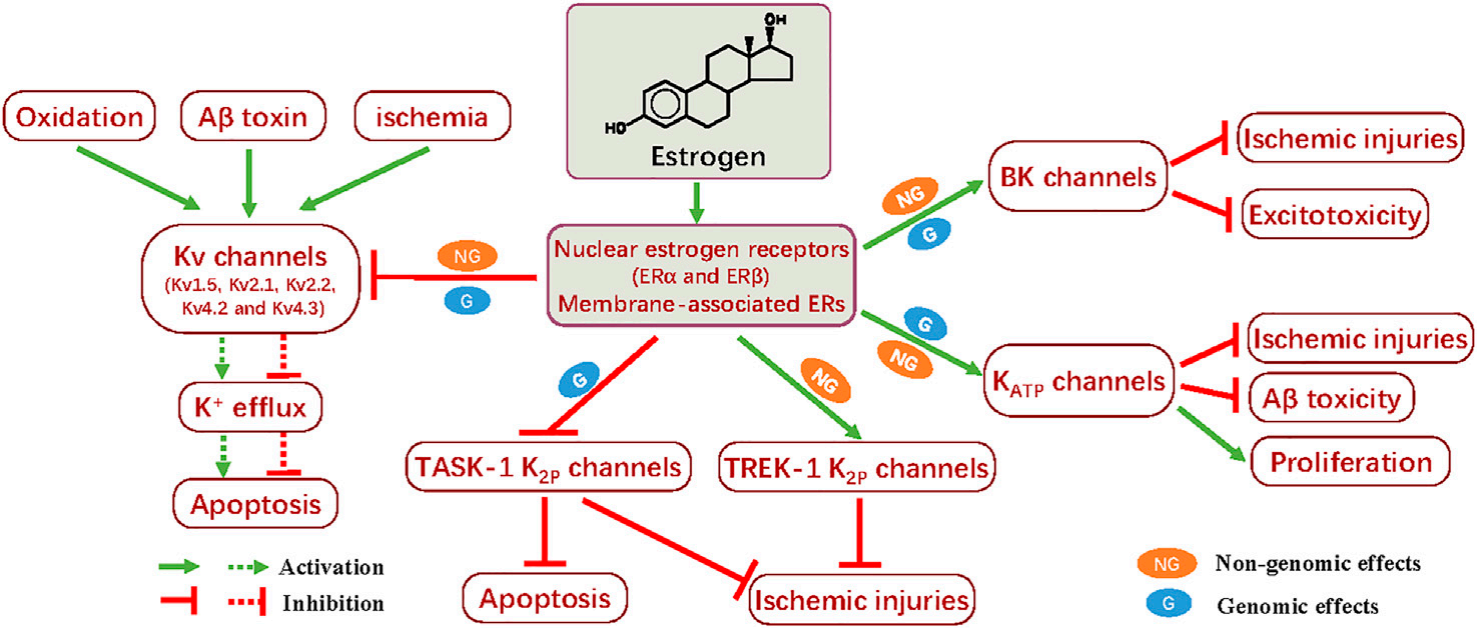
Summary of the neuroprotective effects of estrogens through modulation of potassium channels. Treatment with estrogen generates neuroprotective actions by opposing apoptosis and ischemic injuries; this effect is mediated by the inhibitory impact on the Kv, including Kv1.5, Kv2.1, Kv2.2, Kv4.2, and Kv4.3, and TASK-1 K_2P_ channels. On the other hand, enhanced activity of BK, K_ATP_, and TREK-1 K_2P_ channels induced by estrogen also facilitates the mitigation of ischemic injuries, as well as Aβ toxicity or excitotocity, even promoting cell proliferation. The nuclear estrogen receptors (ERα and ERβ) and membrane-associated ERs participate in the genomic and non-genomic effects, respectively. These distinct approaches could, at least in part, underlie the neuroprotective mechanism of estrogen in modulating K^+^ channels.

Neuronal A-type K^+^ current (I_(A)_) is activated at potentials below the firing threshold of action potentials, with rapidly activating and inactivating kinetics (Johnston et al., 2010). Generally, it is believed that Kv4.2 and Kv4.3 channels are responsible for I_(A)_ in broad areas of the brain, including hippocampal and cortical neurons (Norris and Nerbonne, 2010). The downregulation of Kv4.2, but not Kv4.3, mRNA levels by 17β-estradiol (E2) replacement is observed in the hypothalamic paraventricular nucleus (PVN) neurons (Lee et al., 2013). Nevertheless, Saito et al. (2009)revealed that downregulation of Kv4.3 expression is also conducted by application of estrogens in ventricular myocytes. To date, it appears that estrogens fail to exert an acute non-genomic effect on I_(A)_ (Knock et al., 2001). As mentioned previously, injection of Aβ peptides is able to generate an upregulation of Kv4.2 channel expression in the hippocampus and cerebral cortex, and this event participates in pathogenesis of AD (Pan et al., 2004). As such, the estrogen-mediated inhibition for Kv4.2 and/or Kv4.3 may counteract the Aβ neurotoxic effects on neuronal cells. The involvement of estrogen in regulating Kv channels in neuroprotection can be seen in Figure 1.

## BK channels

Large-conductance Ca^2+^- and voltage-activated K^+^ (BK) channels, encoded by the *Kcnma1* gene, are distributed in diverse tissues, such as neuronal and non-neuronal cells. Normally, they can be activated in response to elevation in intracellular Ca^2+^concentrations and membrane depolarization; in turn, they oppose Ca^2+^ influx and neurotransmitter release, a mechanism for reducing cell excitation (Griguoli et al., 2016). Collected evidence has established a clear neuroprotective role for the opening of BK channels in ischemic brain damage. In acute focal cerebral ischemia, BK channels conduct a beneficial action to limit brain infarction and promote survival, which is also evident in hippocampal slice cultures (Liao et al., 2010), and activation of these channels can contribute to chlorpromazine (CPZ)- and vitamin C-mediated reduction in ischemic brain injury (Li et al., 2014; Li et al., 2019). In hippocampal pyramidal neurons, blockade of BK channels aggravates the cell damage arising from oxygen and glucose deprivation (OGD) (Rundén-Pran et al., 2002). In addition, BK channels predominantly expressed in the spinal cord could generate a protective effect on acute and chronic spinal cord injury (SCI) by improving neuronal functions (Ye et al., 2012; Jacobsen et al., 2018), and the BK channel agonist, isopimaric acid (ISO), mitigates cognitive deficits in 3xTg Alzheimer’s disease (AD) model mice (Wang et al., 2015), indicating obvious and various manners of neuroprotection.

It is well known that BK channels are tetramers of four pore-forming α subunits and four β regulatory subunits, which can be directly activated by estradiol binding to the β subunits (Valverde et al., 1999). Furthermore, several lines of evidence show that estrogens are capable of increasing the expression of BK channels in the different subtypes of neural cells *via* the estrogen receptor β (Nishimura et al., 2008; Li and Qiu, 2015). As a result, elevated activity of BK channels arising from the genomic and/or non-genomic effects of estrogens facilitates their positive influence in cellular survival and proliferation (Coiret et al., 2005). Additionally, 17β-estradiol can activate estrogen receptors to lower the excitotoxicity in ischemia by potentiating the activity of BK channels and decreasing the AMPA/NMDA receptor-mediated spontaneous excitatory postsynaptic currents (sEPSCs), a process that is indicative of the interplay between BK and other ion channels in estrogen-induced neuroprotection (Zhang et al., 2009). The activation of neuroprotective BK channels by 17β-estradiol also leads to an attenuation of neuronal excitability impairment in the ischemia model of oxygen and glucose deprivation (OGD) in rat hippocampal slices (Zhang et al., 2009), which is suggestive of the protective action on memory conferred by estrogen. Taken together, the estrogen-enhanced activity of BK channels facilitates neuroprotection (Figure 1).

## K_ATP_ channels

In addition to regulating the release of pancreatic β-cells, K_ATP_ channels expressed in a number of different brain areas, especially with high levels in the hippocampus and cortex, are thought to be a potential regulator of excitability and neurotransmitter release (Szeto et al., 2018). Neuronal K_ATP_ channels are hetero-octameric complexes consisting of four regulatory subunits SURx and four pore-forming subunits Kir6.x. Activation of K_ATP_ channels conducts the potential effects on mitigating hypoxic-ischemic brain injury, considered as a promising therapeutic target for ischemic stroke (Szeto et al., 2018; Zhao et al., 2021). Remote ischemic preconditioning (RIPC) is capable of lowering the hippocampus damage induced by global cerebral ischemia, which is mediated by the activation of K_ATP_ channels (Mehrjerdi et al., 2015). The elevated neuronal damage after ischemic insults in the hippocampus and neocortex is also observed in the mice lacking Kir6.2-containing K_ATP_ channels (Sun et al., 2006). Overexpression of the SUR1 subunit of K_ATP_ channels in the forebrain opposes seizure induction and excitotoxic damage of hippocampal pyramidal neurons (Hernández-Sánchez et al., 2001). Ketones generate hippocampal synaptic protection in part through increasing the activity of K_ATP_ channels (Kim et al., 2015). Moreover, opening of K_ATP_ channels after application of diazoxide may protect cortical and hippocampal cholinergic neurons against Aβ_1-42_-induced neurotoxicity (Tan et al., 2012). Likewise, sildenafil and diazoxide could reduce the Aβ_25-35_-mediated mitochondrial toxicity through K_ATP_ channels (Son et al., 2018), which is indicative of another protective process arising from this channel. Therefore, K_ATP_ channels in different brain regions, including the hippocampus, may participate in the process of neuroprotection through counteracting ischemia and Aβ neurotoxicity.

Intriguingly, increasing evidence indicates that estrogens also produce a regulatory action in K_ATP_ channels. In GnRH neurons important to the control the female reproductive cycle, 17β-estradiol enhances the activity of K_ATP_ channels *via* a membrane ER-activated PKC-PKA signaling pathway, without affecting the mRNA levels of the Kir6.2 and SUR1 subunits (Zhang et al., 2007; Zhang et al., 2010). However, estrogen-induced upregulation of the Kir6.2 and SUR1 subunits is also observed in brain cortices, opposing focal cerebral ischemia (Zhang et al., 2012). Enhanced expression of the SUR2A subunit of K_ATP_ channels underlies the protective action of estrogen against hypoxia-reoxygenation (Ranki et al., 2002), and estrogen-mediated protection during ischemia-reperfusion stress can be attributed to the elevated expression of the SUR2 subunit (Gao et al., 2014). Moreover, increased expression of K_ATP_ channels after treatment with estrogens exhibits a positive effect on cell proliferation (Park et al., 2008), favoring the neuroprotection of estrogens. Collectively, upregulation of the activity of K_ATP_ channels can contribute to the beneficial effects of estrogens on the nervous system (Figure 1).

## K_2P_ channels

The two-pore-domain background potassium (K_2P_) channels possess a distinct structure known as dimers, each subunit of which comprise two pore-forming loops and four transmembrane (TM) domains (Feliciangeli et al., 2015). These channels remain active over the entire range of membrane potentials owing to the lack of a voltage sensor; as such, they are regarded as “leak” channels (Bayliss and Barrett, 2008). The existence of K_2P_ channels is evident in a variety of cell subtypes, and their function is to stabilize the membrane potential and regulate the excitability and cell volume (Feliciangeli et al., 2015). Of 15 members of the K_2P_ channel family, the stretch- and lipid-activated TREK-1 channels are referred to as the mediator implicated in neuroprotection because of activation by polyunsaturated fatty acids (PUFAs), which occurs against cerebral ischemic insult (Heurteaux et al., 2004). Several K_2P_ channels such as the TREK and TASK channels are located in astrocytes important to brain volume homeostasis, and the ethacrynic acid derivative 4-(2-butyl-6,7-dichloro-2-cyclopentylindan-1-on-5-yl)oxobutyric acid (DCPIB) can inhibit the astroglial swelling and reduce the size of infarct volume during ischemic damage; the neuroprotective effects are thought to arise from activation of TREK-1 channels (Minieri et al., 2013). Choudhury and Sikdar (2018)revealed that 17β-estradiol is able to activate the TREK-1 channels by binding to the G protein-coupled estrogen receptor (GPER), contributing to the neuroprotective effect of this hormone.

The acid- and oxygen-sensitive TASK channel is thought to exert a protective effect on stroke development (Ehling et al., 2015). TASK-1^−/−^ mice show larger infarct volumes in a model of focal cerebral ischemia, and TASK channel inhibition by antagonists increases stroke development (Meuth et al., 2009; Muhammad et al., 2010). Moreover, inactivation of the TASK-1 and TASK-3 channels results in a significant attenuation in the death of cerebellar granule neurons (Lauritzen et al., 2003). These data have established a clear role for TASK channels in counteracting ischemic and apoptotic neuronal death. Chronic applications of 17β-estradiol generate a downregulation of TASK-1 channel expression and improve the proliferation of neuroblastoma N2A cells (Hao and Li, 2014), which is suggestive of the involvement of K_2P_ channel inhibition in estrogen-mediated neuroprotection. Interestingly, both TASK-1 inhibition and TREK-1 activation are capable of participating in the neuroprotective effects conducted by estrogens (Figure 1). More studies are needed to explore the action of estrogens on these background K^+^ channels for the exact underlying mechanism.

## Summary

Collectively, earlier and current data have indicated that estrogens are capable of generating diverse biological actions through multiple approaches and accordingly producing beneficial effects for the symptoms of menopause in women. Many women take estrogen-containing therapies for lots of health-related problems, and clinical trials are also being undertaken to evaluate the outcomes and search for better ways with less adverse actions (Rossouw et al., 2002). The potent neuroprotection conferred by estrogens shows many positive influences to attenuate cognitive aging and brain damage, and extensive research is being conducted to clarify the exact causes for these issues. Clearly, the collected data indicate that many kinds of K^+^ channels, such as Kv, BK, K_ATP_, and K_2P_ channels, also participate in estrogen-mediated actions in nervous tissues to protect against apoptosis, ischemic insult, and Aβ toxicity (Figure 1; Table 1). Interestingly, administration of estrogens produces an inhibitory effect on the Kv and TASK-1 K_2P_ channels but activates the BK, K_ATP_, and TREK-1 K_2P_ channels for a common protective goal. The virtual causes for these phenomena are unknown, and the varying regulation of these K^+^ channels by the genomic or non-genomic actions of estrogens alone or in combination may provide a possible answer. Definitely, additional studies are needed to address these issues. Currently, although the clear role of K^+^ channels in the neuroprotective actions of estrogens is established, experimental data are relatively limited. Accordingly, intensive research needs to be performed to explore the more detailed events of this subject in the future.

**TABLE 1:** Modulation of estrogens on K^+^ channels for neuroprotection.

K^+^ channels	Subtypes	Modulation *via* nuclear ERs (transcription and/or posttranslational level)	Modulation *via* membrane G protein-coupled ERs	Direct modulation
Kv	Kv1.5	Downregulation (Saito et al., 2009; Tsang et al., 2004)	—	No (Wong et al., 2008)
	Kv2.1	Downregulation (El Gebeily and Fiset, 2011)	—	Inhibition (Druzin et al., 2011; Li et al., 2013)
	Kv2.2	—	Inhibition (GPR30-mediated PKC pathway) (Dong et al., 2013)	Inhibition (Druzin et al., 2011)
	Kv4.2	Downregulation Lee et al., 2013)	—	—
	Kv4.3	Downregulation (Saito et al., 2009)	—	—
KCa	BK	Upregulation (Li and Qiu, 2015; Nishimura et al., 2008)	—	Activation (Valverde et al., 1999)
Kir	K_ATP_	Upregulation (Park et al., 2008; Zhang et al., 2012)	Activation (PKC-PKA signaling pathway) (Zhang et al., 2010)	—
K_2P_	TASK-1	Downregulation (Hao and Li, 2014)	—	No (Hao and Li, 2014)
	TREK-1	—	Activation (cAMP-PKA pathway) (Choudhury and Sikdar, 2018)	—
